# Efficacy of different oral H_1_ antihistamine treatments on allergic rhinitis: a systematic review and network meta-analysis of randomized controlled trials

**DOI:** 10.1016/j.bjorl.2023.03.009

**Published:** 2023-04-07

**Authors:** Dongdong Hong, Juanling Weng, Meiting Ye, Yuanxian Liu

**Affiliations:** Shenzhen Traditional Chinese Medicine Hospital, Department of Otorhinolaryngology, People’s Republic of China, Shenzhen, China

**Keywords:** Histamine H_1_ receptor, Antihistamines, Allergic rhinitis, Network meta-analysis

## Abstract

**Introduction:**

Oral H_1_ antihistamines are the first-line treatment for patients with allergic rhinitis, while it is uncertain which kind and dosage of the antihistamines are more effective in improving symptoms of patients.

**Objective:**

To evaluate the efficacy of different oral H_1_ antihistamine treatments on patients with allergic rhinitis by performing a network meta-analysis.

**Methods:**

The search was executed in PubMed, Embase, OVID, the Cochrane Library and ClinicalTrials.gov for relevant studies. The network meta-analysis was performed by using Stata 16.0, and the outcome measures of the analysis were symptom score reductions of patients. Relative risks with 95% Confidence Intervals were used in the network meta-analysis to compare the clinical effect of treatments involved, and Surface Under the Cumulative Ranking Curves (SUCRAs) were also calculated to rank the treatments’ efficacy.

**Results:**

18 eligible randomized controlled studies, involving a total of 9419 participants, were included in this meta-analysis. All the antihistamine treatments outperformed placebo in total symptom score reduction and each individual symptom score reduction. According to the results of SUCRA, rupatadine 20 mg and rupatadine 10 mg were ranked relatively high in reductions of total symptom score (SUCRA: 99.7%, 76.3%), nasal congestion score (SUCRA: 96.4%, 76.4%), rhinorrhea score (SUCRA: 96.6%, 74.6%) and ocular symptom score (SUCRA: 97.2%, 88.8%); rupatadine 20 mg and levocetirizine 5 mg were ranked relatively high in reductions of nasal itching score (SUCRA: 84.8%, 83.4%) and sneezing score (SUCRA: 87.3%, 95.4%); loratadine 10 mg was ranked the lowest in each symptom score reduction besides placebo.

**Conclusion:**

This study suggests that rupatadine is the most effective in alleviating symptoms of patients with allergic rhinitis among different oral H_1_ antihistamine treatments involved, and rupatadine 20 mg performs better than rupatadine 10 mg. While loratadine 10 mg has inferior efficacy for patients to the other antihistamine treatments.

## Highlights

• Rupatadine is the most effective for allergic rhinitis among various oral H_1_ antihistamines.
• Rupatadine 20 mg is more effective for allergic rhinitis than rupatadine 10 mg.
• Loratadine 10 mg has inferior efficacy for allergic rhinitis to the other oral H_1_ antihistamines.

## Introduction

Allergic Rhinitis (AR) is a nasal inflammatory disease characterized by nasal congestion, rhinorrhea, nasal itching, and sneezing, which is usually accompanied by ocular symptoms such as tearing, itching and redness of the eyes. Repeated episodes of symptoms can seriously impair the patients' quality of life and harm their physical and mental health. AR currently affects approximately 40% of the global population,^1^ and the prevalence is still increasing in most countries and regions. The oral H_1_ antihistamines are strongly recommended by guidelines for the first-line treatment of AR,^2^ and actually, they are used quite frequently in clinical treatment. However, it is often unclear which kind and dosage of the antihistamines can improve the symptoms of AR patients more effectively, leaving many physicians to choose antihistamines based on their own experience. In this review, the improvement of different oral H_1_ antihistamine treatments on AR patients’ symptoms was compared through a network meta-analysis and systematic review, in order to find out the treatment with better efficacy and provide some references for clinical decision making.

## Methods

### Search strategy

This network meta-analysis and systematic review, begun in December 2021 and completed in May 2022, taking approximately 5 months, were conducted, and reported in accordance with the Preferred Reporting Items for Systematic Reviews and Meta-Analyses (PRISMA) 2020.^3^ The search was performed by two reviewers using PubMed, Embase, OVID, the Cochrane Library and ClinicalTrials.gov with a search period from the creation date of each database to April 2022. The keywords used in the search strategy included “allergic rhinitis”, “allergic rhinitides”, “hay fever”, “antihistamine”, “histamine antagonist”, “loratadine”, “desloratadine”, “cetirizine”, “levocetirizine”, “rupatadine”, “fexofenadine”, “ebastine”, “bilastine”, “terfenadine”, “olopatadine”, “acrivastine”. The reference lists of the obtained literature were also searched to ensure that no available studies were missed.

### Inclusion and exclusion criteria

Studies included in this review were required to meet the following criteria: (1) The type of studies was randomized controlled trial; (2) The content of studies was the comparison among different oral H_1_ antihistamine treatments or between oral H_1_ antihistamine treatment and placebo in patients with AR, whose age, gender, race and disease type of patients are not limited; (3) The reductions of patients’ symptom scores, which included Total Symptom Score (TSS), nasal congestion score, rhinorrhea score, nasal itching score, sneezing score and/or ocular symptom (tearing/itching/redness) score, after treatment were provided or could be calculated as the outcome measures in each study.

Studies meeting the following criteria were excluded: (1) The studies were reported in case report format or review article format; (2) The data of studies were missing, or the data could not be extracted from the published results, or the raw data could not be obtained.

### Data extraction and risk of bias assessment

All data were extracted from the involved studies by two reviewers separately and cross-checked afterwards. The extracted data included mean and Standard Deviation (SD) of the reductions in symptom scores after treatment, as well as the number, age, gender, and disease type of patients in each intervention group. The GetData Graph Digitizer software (version 2.26) was used to extract data in figures. The data reported with Standard Error (SE) or 95% Confidence Interval (95% CI) were converted to SD referring to the Cochrane Handbook for Systematic Reviews of Interventions (version 6.2).^4^ The risk of material bias of the studies was assessed by using the Cochrane risk-of-bias tool for randomized trials (version 2),^5^ and each study was assessed as “low risk of bias”, “some concerns” or “high risk of bias” in each domain.

### Statistical analysis

Meta-analysis was performed by using Stata 16.0. The net league command was used to calculated Relative Risk (RR) with 95% CI between two treatments. For ranking all the treatments, the Surface Under the Cumulative Ranking Curve (SUCRA) from 0% to 100% was applied, with 0% being the statistically worst and 100% being the statistically best.^6^ The inconsistency between direct and indirect evidence was evaluated by the loop-specific method to reflect heterogeneity in each loop. Inconsistency indicated difference between direct and indirect evidence with 95% CI for Inconsistency Factor (IF) excluding 0. The potential publication bias was assessed by comparison-adjusted funnel plots.

## Results

**Figure 1:**
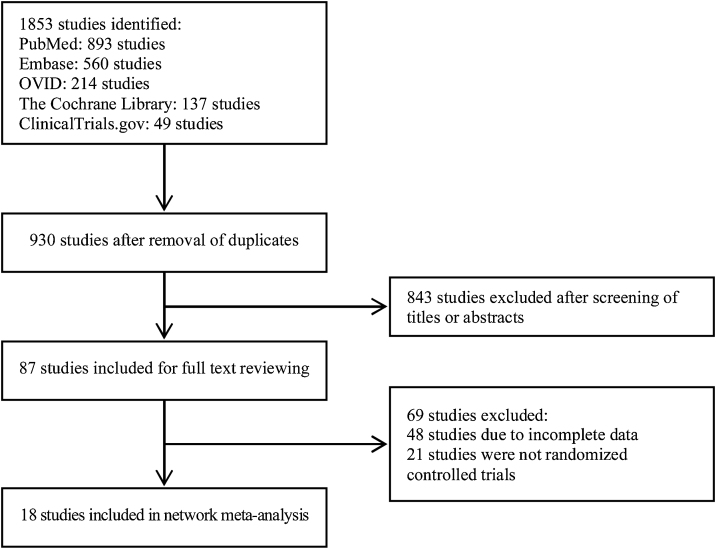
Flowchart of the selection process.

In total, 1853 studies were identified based on the search strategy. After screening studies according to the inclusion and exclusion criteria and removing duplicates, 18 articles were included in this meta-analysis finally. The selection process and result are shown in Fig. 1, and characteristics of the included studies are summarized in Table 1.^7^, ^8^, ^9^, ^10^, ^11^, ^12^, ^13^, ^14^, ^15^, ^16^, ^17^, ^18^, ^19^, ^20^, ^21^, ^22^, ^23^, ^24^

**Table 1:** Characteristics of the included studies.

Study	Year	Country	Overall risk of bias	Type of AR	Follow-up	Group (daily dosage)	Number	Mean age (y)	Gender (m/f)
Kowalski	2009	Poland	High risk	PAR	4 weeks	Rupatadine 10 mg	73	33.3 ± 11.3	30/43
						Rupatadine 20 mg	71	28.6 ± 10.4	28/43
						Loratadine 10 mg	70	29.6 ± 10.7	36/34
						Placebo	69	26.0 ± 9.7	40/29
Marmouz	2011	France	High risk	PAR	4 weeks	Rupatadine 10 mg	65	31.4	18/47
						Rupatadine 20 mg	68	33.8	26/42
						Cetirizine 10 mg	66	32.2	28/38
						Placebo	70	30.9	27/43
Okubo	2019	Japan	Some concerns	SAR	2 weeks	Rupatadine 10 mg	298	37.5 ± 13.0	158/140
						Rupatadine 20 mg	300	36.8 ± 13.0	153/147
						Placebo	302	35.8 ± 13.1	146/156
Lukat	2013	Germany	High risk	SAR	4 weeks	Rupatadine 10 mg	117	30.8 ± 11.2	61/56
						Desloratadine 5 mg	117	32.0 ± 12.5	52/65
						Placebo	122	31.8 ± 12.6	66/56
Fantin	2008	Argentina	High risk	PAR	12 weeks	Rupatadine 10 mg	183	28.58 ± 13.48	59/124
						Cetirizine 10 mg	174	29.18 ± 12.75	73/101
						Placebo	185	30.13 ± 12.48	56/129
Molina	2010	Spain	High risk	PAR	4 weeks	Rupatadine 10 mg	69	27.0 ± 9.6	30/39
						Ebastine 10 mg	77	27.0 ± 10.2	35/42
						Placebo	73	29.0 ± 10.1	39/34
Davies	1998	UK	High risk	PAR	4 weeks	Ebastine 10 mg	103	31.6	59/44
						Ebastine 20 mg	111	32.8	53/58
						Loratadine 10 mg	103	31.2	49/54
Hampel	2004	USA	High risk	SAR	4 weeks	Ebastine 10 mg	188	38.2 ± 12.5	99/89
						Ebastine 20 mg	186	37.9 ± 13.4	85/101
						Loratadine 10 mg	189	37.3 ± 13.6	87/102
						Placebo	186	37.0 ± 13.5	93/93
Ratner	2004	USA	High risk	SAR	4 weeks	Ebastine 20 mg	282	38.0 ± 13.7	111/171
						Loratadine 10 mg	279	38.9 ± 13.8	108/171
						Placebo	142	37.5 ± 14.7	53/89
Casale	1999	USA	High risk	SAR	2 weeks	Fexofenadine 120 mg	287	32 ± 12	105/182
						Fexofenadine 180 mg	282	33 ± 12	98/184
						Placebo	292	32 ± 11	101/191
Howarth	1999	UK	High risk	SAR	2 weeks	Fexofenadine 120 mg	211	33	117/94
						Fexofenadine 180 mg	202	32	100/102
						Cetirizine 10 mg	207	33	100/107
						Placebo	201	34	103/98
Van	2000	Belgium	High risk	SAR	2 weeks	Fexofenadine 120 mg	232	30.9 ± 11.51	101/131
						Loratadine 10 mg	228	31.9 ± 12.22	101/127
						Placebo	225	30.6 ± 12.14	104/121
Bachert	2004	Belgium	Some concerns	PAR	4 weeks	Levocetirizine 5 mg	278	29.8 ± 8.9	126/152
						Placebo	273	30.8 ± 8.8	115/158
Guilemany	2012	Spain	High risk	PAR	4 weeks	Levocetirizine 5 mg	14	31.1 ± 10.9	7/7
						Placebo	13	32.6 ± 10.1	7/6
Noonan	2003	USA	High risk	SAR	2 weeks	Cetirizine 10 mg	202	35.8 ± 10.6	63/139
						Placebo	198	37.4 ± 11.1	72/126
Pradalier	2007	France	High risk	SAR	2 weeks	Desloratadine 5 mg	234	32.7 ± 10.7	121/113
						Placebo	249	32.4 ± 11.0	129/120
Bousquet	2009	France	High risk	IAR	2 weeks	Desloratadine 5 mg	276	33.8 ± 12.0	121/155
						Placebo	271	34.6 ± 12.8	106/165
Bousquet	2013	France	High risk	PAR	12 weeks	Desloratadine 5 mg	355	NA	NA
						Placebo	351	NA	NA

AR, Allergic Rhinitis; PAR, Perennial Allergic Rhinitis; SAR, Seasonal Allergic Rhinitis; IAR, Intermittent Allergic Rhinitis.

### Network meta-analysis

**Figure 2:**
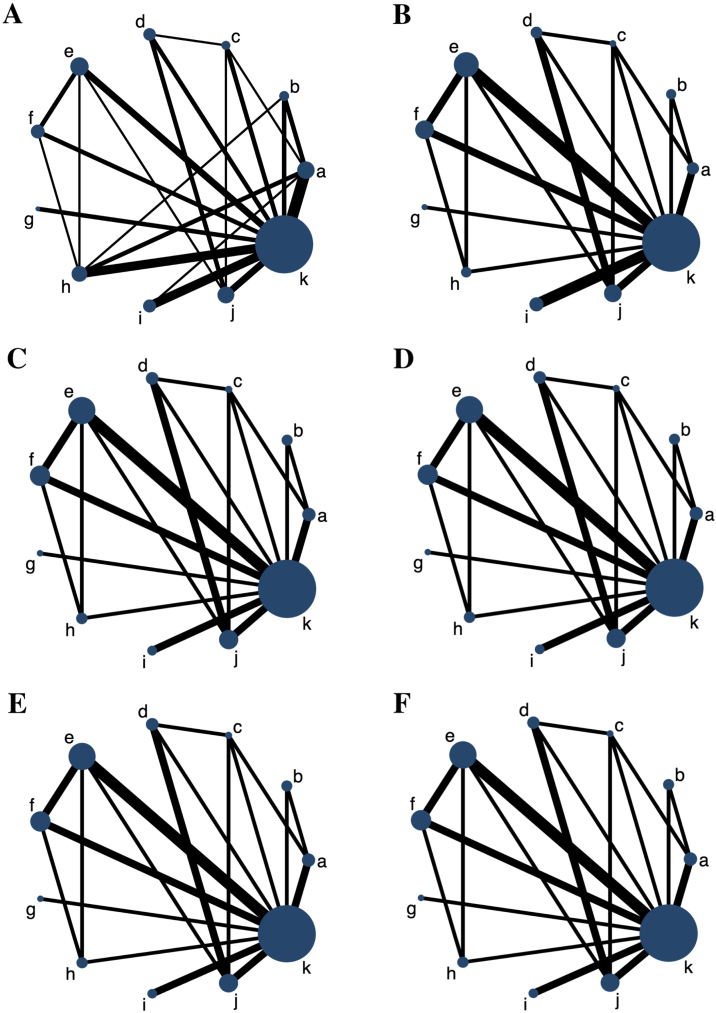
Network plots for all the outcome measures. The node sizes are weighted by the sample of treatments, and the line widths are weighted by the number of studies involved. (A) Total Symptom Score (TSS) reduction; (B) Nasal congestion score reduction; (C) Rhinorrhea score reduction; (D) Nasal itchinig score reduction; (E) Sneezing score reduction; (F) Ocular symptom score reduction. (a) Rupatadine 10 mg; (b) Rupatadine 20 mg; (c) Ebastine 10 mg; (d) Ebastine 20 mg; (e) Fexofenadine 120 mg; (f) Fexofenadine 180 mg; (g) Levocetirizine 5 mg; (h) Cetirizine 10 mg; (i) Desloratadine 5 mg; (j) Loratadine 10 mg; (k) Placebo.

6 networks involving 6 major outcome measures were established, and each network plot involved 10 antihistamine treatments and placebo. The network plots for all the outcome measures are shown in Fig. 2. The number of studies on rupatadine 10 mg was the largest among all the active treatments.

### Summary of treatment effect for TSS reduction

**Figure 3:**
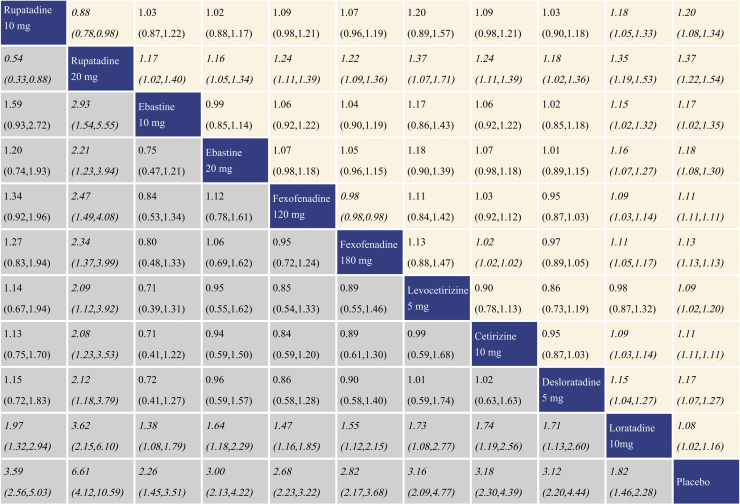
Network meta-analysis results for Total Symptom Score (TSS) reduction and nasal congestion score reduction. The data in the boxes are Relative Risks (RRs) with 95% Confidence Intervals. The lower left triangular area represents the results for TSS reduction, and RR > 1 suggests that the corresponding column treatment is superior to the corresponding row treatment, and the opposite is true for RR < 1. The upper right triangular area represents the results for nasal congestion score reduction, and RR > 1 suggests that the corresponding row treatment is superior to the corresponding column treatment, and the opposite is true for RR < 1. Statistically significant results are shown in italic.

With regard to TSS reduction, all the antihistamine treatments performed better than placebo, and the effect size was the largest for rupatadine 20 mg and rupatadine 10 mg. In comparisons among the antihistamine treatments, rupatadine 20 mg was more effective than the others, and in contrast, loratadine 10 mg was inferior to the other treatments. The network meta-analysis results for TSS reduction are presented in the lower left triangular area of Fig. 3.

### Summary of treatment effect for nasal congestion score reduction

With regard to nasal congestion score reduction, all the antihistamine treatments were superior to placebo, and the effect size was the largest for rupatadine 20 mg and rupatadine 10 mg. In comparisons among the antihistamine treatments, rupatadine 20 mg performed better than the others. Fexofenadine 180 mg was more effective than fexofenadine 120 mg, cetirizine 10 mg and loratadine 10 mg. There was no significant difference in efficacy between levocetirizine 5 mg and loratadine 10 mg, while loratadine 10 mg was inferior to the other treatments. The network meta-analysis results for nasal congestion score reduction are presented in the upper right triangular area of Fig. 3.

### Summary of treatment effect for rhinorrhea score reduction

**Figure 4:**
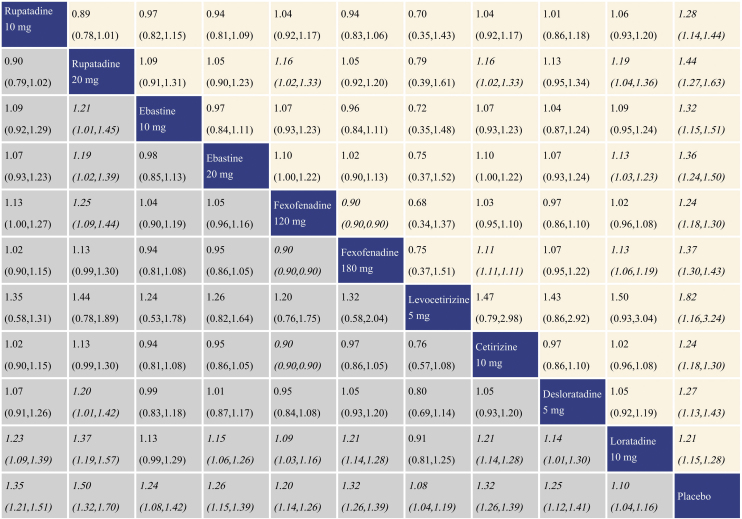
Network meta-analysis results for rhinorrhea score reduction and nasal itching score reduction. The data in the boxes are Relative Risks (RRs) with 95% Confidence Intervals. The lower left triangular area represents the results for rhinorrhea score reduction, and RR > 1 suggests that the corresponding column treatment is superior to the corresponding row treatment, and the opposite is true for RR < 1. The upper right triangular area represents the results for nasal itching score reduction, and RR > 1 suggests that the corresponding row treatment is superior to the corresponding column treatment, and the opposite is true for RR < 1. Statistically significant results are shown in italic.

With regard to rhinorrhea score reduction, all the antihistamine treatments performed better than placebo, and the effect size was the largest for rupatadine 20 mg and rupatadine 10 mg. In comparisons among the antihistamine treatments, rupatadine 20 mg was more effective than ebastine 10 mg, ebastine 20 mg, fexofenadine 120 mg, desloratadine 5 mg and loratadine 10 mg. Both of fexofenadine 180 mg and cetirizine 10 mg performed better than fexofenadine 120 mg and loratadine 10 mg. Rupatadine 10 mg, ebastine 20 mg, fexofenadine 120 mg and desloratadine 5 mg also showed better effect than loratadine 10 mg. The network meta-analysis results for rhinorrhea score reduction is presented in the lower left triangular area of Fig. 4.

### Summary of treatment effect for nasal itching score reduction

With regard to nasal itching score reduction, each antihistamine treatment was superior to placebo, and the effect size was the largest for rupatadine 20 mg and rupatadine 10 mg. In comparisons among the antihistamine treatments, both of rupatadine 20 mg and fexofenadine 180 mg performed better than fexofenadine 120 mg, cetirizine 10 mg and loratadine 10 mg. Ebastine 20 mg also performed better than loratadine 10 mg. The network meta-analysis results for nasal itching score reduction are presented in the upper right triangular area of Fig. 4.

### Summary of treatment effect sneezing score reduction

**Figure 5:**
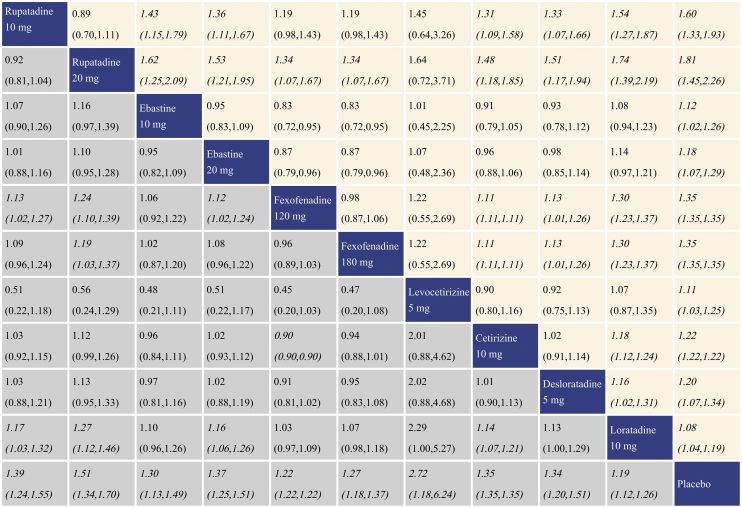
Network meta-analysis results for sneezing score reduction and ocular symptom score reduction. The data in the boxes are Relative Risks (RRs) with 95% Confidence Intervals. The lower left triangular area represents the results for sneezing score reduction, and RR > 1 suggests that the corresponding column treatment is superior to the corresponding row treatment, and the opposite is true for RR < 1. The upper right triangular area represents the results for ocular symptom score reduction, and RR > 1 suggests that the corresponding row treatment is superior to the corresponding column treatment, and the opposite is true for RR < 1. Statistically significant results are shown in italic.

With regard to sneezing score reduction, all the antihistamine treatments were superior to placebo, and the effect size was the largest for levocetirizine 5 mg and rupatadine 20 mg. In comparisons among the antihistamine treatments, rupatadine 10 mg, rupatadine 20 mg and ebastine 20 mg were more effective than fexofenadine 120 mg and loratadine 10 mg; moreover, rupatadine 20 mg was more effective than fexofenadine 180 mg. Cetirizine 10 mg was superior to fexofenadine 120 mg and loratadine 10 mg. The network meta-analysis results for sneezing score reduction are presented in the lower left triangular area of Fig. 5.

### Summary of treatment effect for ocular symptom score reduction

With regard to ocular symptom score reduction, all the antihistamine treatments performed better than placebo, and the effect size was the largest for rupatadine 20 mg and rupatadine 10 mg. In comparisons among the antihistamine treatments, rupatadine 10 mg was more effective than ebastine 10 mg, ebastine 20 mg, cetirizine 10 mg, desloratadine 5 mg and loratadine 10 mg. Rupatadine 20 mg was superior to the other treatments except rupatadine 10 mg and levocetirizine 5 mg. Both of fexofenadine 120 mg and fexofenadine 180 mg performed better than cetirizine 10 mg, desloratadine 5 mg and loratadine 10 mg. Cetirizine 10 mg and desloratadine 5 mg performed better than loratadine 10 mg. The network meta-analysis results for ocular symptom score reduction are presented in the upper right triangular area of Fig. 5.

### Ranking of the treatments by SUCRA

**Figure 6:**
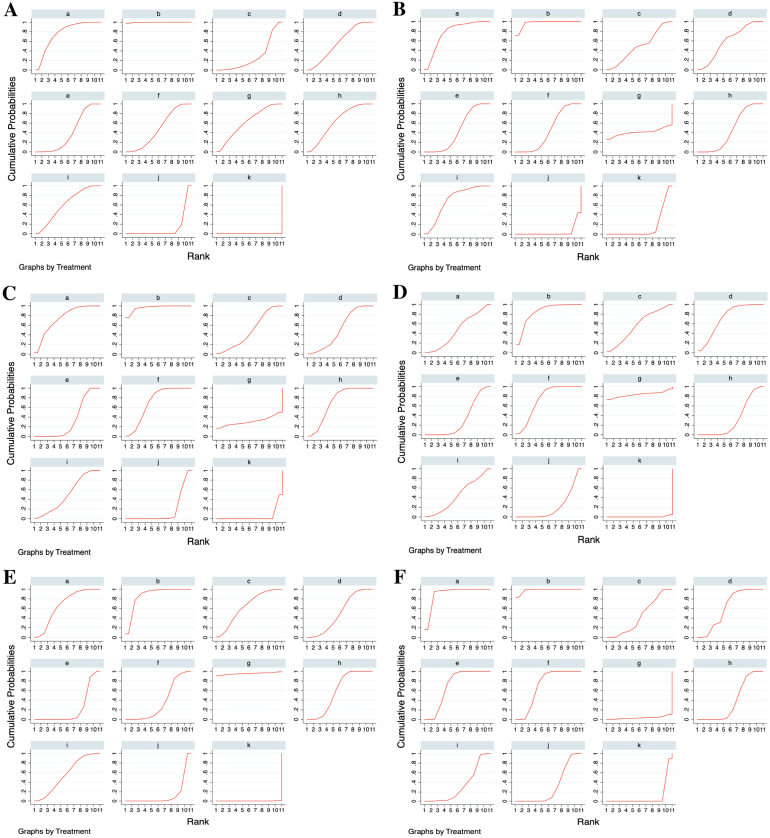
Surface Under the Cumulative Ranking Curves (SUCRAs) of the treatments for all the outcome measures. (A) Total Symptom Score (TSS) reduction; (B) Nasal congestion score reduction; (C) Rhinorrhea score reduction; (D) Nasal itchinig score reduction; (E) Sneezing score reduction; (F) Ocular symptom score reduction. (a) Rupatadine 10 mg; (b) Rupatadine 20 mg; (c) Ebastine 10 mg; (d) Ebastine 20 mg; (e) Fexofenadine 120 mg; (f) Fexofenadine 180 mg; (g) Levocetirizine 5 mg; (h) Cetirizine 10 mg; (i) Desloratadine 5 mg; (j) Loratadine 10 mg; (k) Placebo.

The treatments were ranked for all the outcome measures according to their SUCRAs, as shown in Table 2; the SUCRA of each treatment is presented in Fig. 6. Rupatadine 20 mg and rupatadine 10 mg were the top 2 treatments for reductions of TSS (SUCRA: 99.7%, 76.3%), nasal congestion score (SUCRA: 96.4%, 76.4%), rhinorrhea score (SUCRA: 96.6%, 74.6%) and ocular symptom score (SUCRA: 97.2%, 88.8%); rupatadine 20 mg and levocetirizine 5 mg were the top 2 treatments for reductions of nasal itching score (SUCRA: 84.8%, 83.4%) and sneezing score (SUCRA: 87.3%, 95.4%); loratadine 10 mg was ranked the lowest in all the symptom score reductions besides placebo.

**Table 2:** Network meta-analysis results for ranking of the treatments on all the outcome measures.

	TSS reduction			Nasal congestion score reduction			Rhinorrhea score reduction			Nasal itching score reduction			Sneezing score reduction			Ocular symptom score reduction		
Treatment	SUCRA (%)	PrBest (%)	Mean rank	SUCRA (%)	PrBest (%)	Mean rank	SUCRA (%)	PrBest (%)	Mean rank	SUCRA (%)	PrBest (%)	Mean rank	SUCRA (%)	PrBest (%)	Mean rank	SUCRA (%)	PrBest (%)	Mean rank
Rupatadine 10 mg	76.3	0.6	3.4	73.4	0.8	3.7	74.6	2.9	3.5	47.1	0.4	6.3	66.5	0.4	4.4	88.8	12.8	2.1
Rupatadine 20 mg	99.7	97.5	1.0	96.4	68.3	1.4	96.6	75.1	1.3	84.8	18.1	2.5	87.3	7.4	2.3	97.2	76.1	1.3
Ebastine 10 mg	27.9	0.0	8.2	62.0	2.5	4.8	47.4	1.2	6.3	57.3	2.7	5.3	45.1	0.2	6.5	28.6	0.0	8.1
Ebastine 20 mg	57.0	0.3	5.3	69.2	1.1	4.1	52.7	0.6	5.7	72.1	3.7	3.8	65.7	0.6	4.4	39.9	0.0	7.0
Fexofenadine 120 mg	41.1	0.0	6.9	35.1	0.0	7.5	33.8	0.0	7.6	32.5	0.0	7.7	22.7	0.0	8.7	72.0	0.0	3.8
Fexofenadine 180 mg	49.1	0.0	6.1	53.6	0.0	5.6	70.9	0.7	3.9	73.6	2.1	3.6	37.1	0.0	7.3	72.1	0.0	3.8
Levocetirizine 5 mg	62.0	1.0	4.8	40.5	26.8	6.9	30.9	17.5	7.9	83.4	72.4	2.7	95.4	91.0	1.5	38.2	11.1	7.2
Cetirizine 10 mg	63.5	0.2	4.6	35.1	0.0	7.5	71.0	0.8	3.9	32.4	0.0	7.8	59.4	0.0	5.1	48.9	0.0	6.1
Desloratadine 5 mg	61.4	0.5	4.9	64.2	0.5	4.6	50.6	1.1	5.9	44.5	0.7	6.6	56.8	0.4	5.3	43.6	0.0	6.6
Loratadine 10 mg	12.0	0.0	9.8	12.8	0.0	9.7	16.5	0.0	9.3	21.8	0.0	8.8	14.0	0.0	9.6	14.9	0.0	9.5
Placebo	0.0	0.0	11.0	7.7	0.0	10.2	5.1	0.0	10.5	0.5	0.0	11.0	0.1	0.0	11.0	5.6	0.0	10.4

TSS, Total Symptom Score; SUCRA, Surface Under the Cumulative Ranking Curve; PrBest, Probability of Being the best.

### Inconsistency

**Figure 7:**
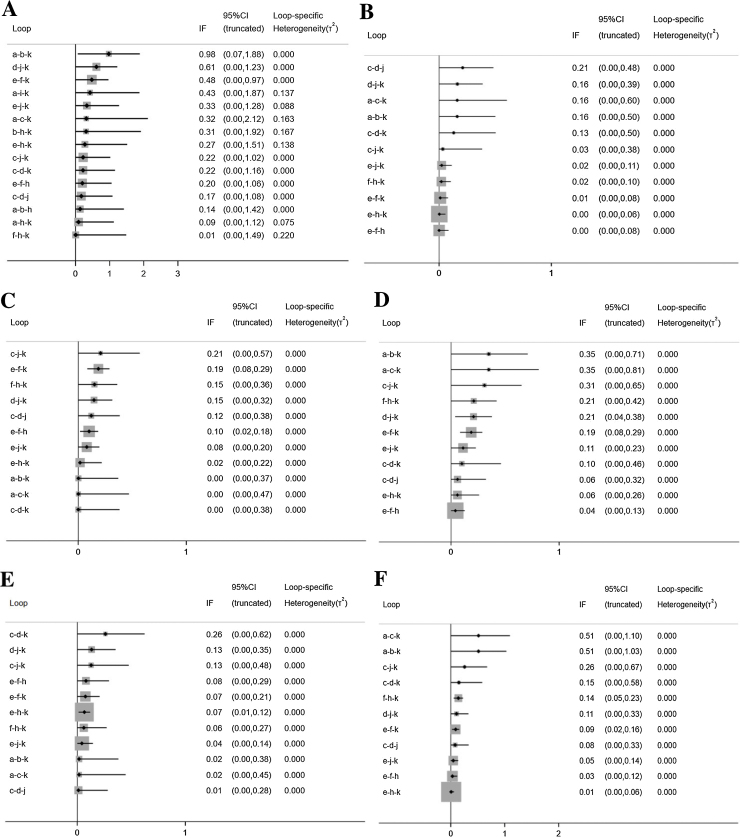
Inconsistency in closed loops for all the outcome measures. IF, Inconsistency Factor; CI, Confidence Interval; (A) Total Symptom Score (TSS) reduction; (B) Nasal congestion score reduction; (C) Rhinorrhea score reduction; (D) Nasal itchinig score reduction; (E) Sneezing score reduction; (F) Ocular symptom score reduction. (a) Rupatadine 10 mg; (b) Rupatadine 20 mg; (c) Ebastine 10 mg; (d) Ebastine 20 mg; (e) Fexofenadine 120 mg; (f) Fexofenadine 180 mg; (g) Levocetirizine 5 mg; (h) Cetirizine 10 mg; (i) Desloratadine 5 mg; (j) Loratadine 10 mg; (k) Placebo.

The inconsistency plots are shown in Fig. 7. Almost all the 95% CIs for their respective IF contained 0, which indicated that there was no significant inconsistency across all the closed loops, and the conclusions of consistency model were reliable.

### Publication bias

**Figure 8:**
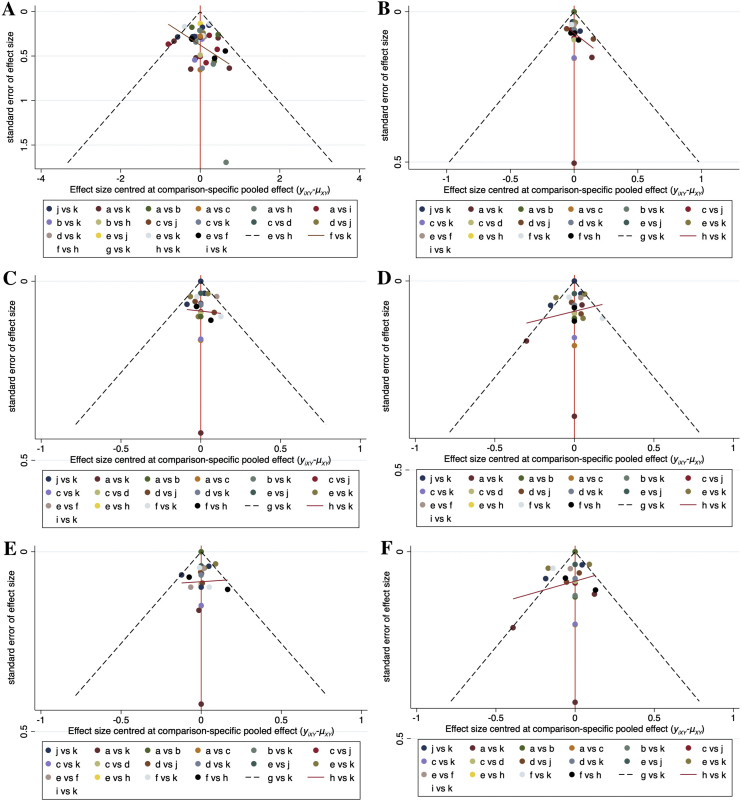
Comparison-adjusted funnel plots for all the outcome measures. (A) Total Symptom Score (TSS) reduction; (B) Nasal congestion score reduction; (C) Rhinorrhea score reduction; (D) Nasal itchinig score reduction; (E) Sneezing score reduction; (F) Ocular symptom score reduction. (a) Rupatadine 10 mg; (b) Rupatadine 20 mg; (c) Ebastine 10 mg; (d) Ebastine 20 mg; (e) Fexofenadine 120 mg; (f) Fexofenadine 180 mg; (g) Levocetirizine 5 mg; (h) Cetirizine 10 mg; (i) Desloratadine 5 mg; (j) Loratadine 10 mg; (k) Placebo.

Comparison-adjusted funnel plots for the outcome measures in the network meta-analysis are shown in Fig. 8. Scatters of the same color were not completely symmetrical in all plots, which meant that publication bias may exist for the outcome measures involved.

## Discussion

Histamine is an important inflammatory mediator in the pathogenetic process of AR, which can cause nasal itching and paroxysmal sneezing by acting on H_1_ receptors on sensory nerve endings in nasal cavity.^25^ In addition, it has a strong vasodilatory effect, which can increase the permeability of capillaries and venules, and then plasma leaks into the nasal tissue, causing local tissue edema and eventually leading to nasal congestion and rhinorrhea. Oral H_1_ antihistamines are able to bind the histamine H_1_ receptors and thereby block the action of histamine. They have been prescribed to patients since the first clinically useful antihistamine, phenbenzamine/pyribenzamine, was developed in 1942. However, due to the central nervous system toxicity of the first-generation antihistamines, the clinical application of antihistamines was significantly limited at the beginning. Until non-sedative terfenadine was first used clinically in the United States in 1985, antihistamines were gradually widely applied in the treatment of allergic diseases.^26^ In a retrospective survey on AR medication, the prescription rate of antihistamines in 2018 was as high as 79.75%, significantly higher than that of glucocorticoids,^27^ which implied the frequent use of antihistamines in AR treatment. The oral H_1_ antihistamines for treating AR currently mainly include loratadine, desloratadine, cetirizine, levocetirizine, ebastine, fexofenadine, rupatadine, etc. Despite their variety, it remains unclear for many physicians which kind and dosage are more effective for AR patients, and thus we performed this network meta-analysis to rank different oral H_1_ antihistamine treatments.

The network meta-analysis results demonstrated that rupatadine 20 mg and rupatadine 10 mg were the most effective for improving overall symptoms, nasal congestion, rhinorrhea, and ocular symptoms in AR patients among all the antihistamine treatments involved. And for improvement of sneezing and nasal itching, rupatadine 20 mg and levocetirizine 5 mg were superior to the other treatments. Histamine is not the only inflammatory mediator that causes AR and therefore a kind of antihistamine which can inhibit more inflammatory mediators may perform better in relieving AR symptoms. As the strongest inflammatory mediator known to increase vascular permeability, potent Platelet-Activating Factor (PAF) can cause edema and exudation of nasal mucosa, resulting in nasal congestion and discharge.^28^ Moreover, the chemotactic effect of PAF on inflammatory cells can make eosinophils accumulate in nasal tissue, thereby aggravating the nasal inflammation.^29^ In addition to being an H_1_ antagonist, rupatadine is also a PAF inhibitor, which can simultaneously inhibit the effect of histamine and PAF in AR. This may be the reason why rupatadine obtained better effect than the other antihistamines for alleviating AR symptoms, so we recommend rupatadine as the first choice for patients with AR if physicians are able to prescribe it. Furthermore, given that the efficacy of rupatadine 20 mg was better than that of rupatadine 10 mg, the former dosage may be preferable for AR patients. Certainly, for patients with nasal itching or sneezing as their main complaints, levocetirizine 5 mg can also be prescribed to them for better efficacy based on the results of this study.

There were also comparisons between two dosages of ebastine and fexofenadine separately in this study. For ebastine, 20 mg dosage was ranked higher than 10 mg dosage in all the symptom score reductions. For fexofenadine, the rankings of 120 mg dosage and 180 mg dosage were almost the same in ocular symptom reduction, while the latter were higher in the other score reductions. Therefore, we recommend that 20 mg dosage should be preferred over 10 mg dosage when using ebastine in treatment of AR; for the use of fexofenadine, both of 120 mg and 180 mg dosage can be selected for patients with relatively obvious ocular symptoms, while for the other patients, 180 mg dosage should be selected preferentially. In terms of rankings, loratadine was the least effective for AR patients among the antihistamines involved, so if oral antihistamines are to be prescribed to AR patients, other available antihistamines should probably be choosed first rather than loratadine.

There were some limitations in our study. Firstly, the small number of included studies may reduce the reliability of our conclusions. Secondly, due to the lack of detailed descriptions on randomization, allocation concealment and blinding, most literature included was at high risk of bias, resulting in the low quality of evidences in this study. Thirdly, studies containing some other antihistamines such as terfenadine, olopatadine and bilastine were filtered out during the selection process, which limited us from comparing more kinds of antihistamines. Finally, although nearly all the studies reported adverse events, most of these adverse events could not be identified as treatment-related, and most of the studies reported the number of events rather than the number of patients, so network meta-analysis on comparing the safety of different oral antihistamines was unable to be performed. Thus, more high-quality, large-sample and standard randomized controlled trials are necessary to be conducted for obtaining more clinical evidence.

## Conclusion

This study suggests that rupatadine is the most effective in treatment of AR patients among the multiple oral H_1_ antihistamines involved, and rupatadine 20 mg performs better than rupatadine 10 mg. While loratadine 10 mg has inferior efficacy for AR patients to the other antihistamines, which is the oral antihistamine treatment we least recommend. For the other kinds and dosages of antihistamines, physicians can choose based on the clinical manifestations of patients and the results of treatment rankings in this study.

## Funding

This study did not receive any specific grant from funding agencies in the public, commercial, or not-for-profit sectors.

## Conflicts of interest

The authors declare no conflicts of interest.
